# Trichoscopic Evaluation of the Effectiveness of Topical Stemoxydine for Hair Restoration in Androgenetic Alopecia: A Prospective Pre–Post Single‐Arm Exploratory Study

**DOI:** 10.1111/jocd.70768

**Published:** 2026-03-30

**Authors:** Yasmina Ahmed Elattar, Noha Nabil Doghem, Mai Tarek Amin, Soha Abdalla Hawwam

**Affiliations:** ^1^ Dermatology and Venereology Department, Faculty of Medicine Tanta University Tanta Egypt

**Keywords:** androgenetic alopecia, hypoxia‐inducible factor, kenogen phase, Stemoxydine

## Abstract

**Background:**

Androgenetic Alopecia (AGA) is a prevalent hereditary disorder leading to hair loss. Stemoxydine, a competitive (Prolyl 4‐hydroxylase) inhibitor, may activate Hypoxia‐Inducible Factor 1‐alpha signaling and shorten the hair kenogen phase, potentially increasing hair density as a proposed treatment for AGA.

**Aims:**

This study aimed to evaluate the effectiveness of topical Stemoxydine in the treatment of AGA.

**Patients and Methods:**

The study included 35 patients with AGA treated with a topical solution of Stemoxydine twice daily for 3 months. Clinical outcomes were evaluated at 6 and 12 weeks using clinical photography, dermoscopy, and digital dermoscopy. The primary endpoint was the percentage of clinical improvement after 12 weeks of treatment application. Dermoscopy and digital image analysis were performed to assess hair growth, density, and diameter. Subgroup analysis compared outcomes between males and females. Adverse events were recorded and evaluated for severity and tolerability.

**Results:**

A total of 35 participants (23 females, 12 males) aged 19–45 years (mean ± SD: 29.2 ± 6.98) were enrolled. The mean age was 31.1 ± 7.28 years for females and 25.7 ± 4.87 years for males. A positive family history of alopecia was reported in 62.9% of participants. Baseline alopecia severity included Grade 2 in 25.7%, Grade 3 in 45.7%, and Grade 4 in 28.6% of cases. After 3 months of treatment application, we observed a median clinical improvement of 80% (range: 35%–100%). As per the Quartile Grading Scale, 57.1% of patients demonstrated significant improvement, while 34.3% showed moderate improvement. Statistically significant improvements in AGA grades were observed post‐treatment. The dermoscopic evaluation revealed new hair growth in 97.1% of patients by 1.5 months, with digital analysis demonstrating significant increases in both hair density (*p* < 0.001) and diameter (*p* < 0.001). Subgroup analysis indicated males achieved significantly more significant clinical improvement (*p* < 0.001), increased hair density (*p* < 0.001), and larger diameter (*p* = 0.02) compared to females. Adverse events were mild and well‐tolerated, with the most common being redness (22.9%).

**Conclusion:**

In conclusion, topical Stemoxydine demonstrated significant clinical benefits in treating AGA, enhancing hair density with minimal adverse effects.

**Abbreviations**
5α‐reductase: 5‐alpha reductase
AGA: Androgenetic alopecia
cm2: Square centimeters
DHT: Dihydrotestosterone
DPCs: Dermal papilla cells
FDA: Food and Drug Administration
HIF‐1α: Hypoxia‐Inducible Factor 1‐alpha
IQR: Interquartile Range
mm: Millimeters
*n* (%): data presented as frequency and percentage
P4H: Prolyl‐4‐hydroxylase
SD: Standard Deviation
SPSS: Statistical Package for the Social Sciences

## 1 Introduction

Androgenetic Alopecia (AGA), also known as male or female pattern baldness, is a genetic disorder characterized by progressive loss of terminal hair of the scalp [1]. It exhibits a distinct distribution pattern in both genders, with males typically manifesting hair loss at the vertex and frontotemporal regions [2]. In contrast, females commonly retain their frontal hairline while experiencing diffuse hair loss at the crown and top of the head [3]. This condition is considered the most prevalent type of alopecia [4]. It may manifest at any age after puberty, significantly impacting the individuals' quality of life negatively [5, 6].

Despite the availability of several medical, light‐based, and complementary treatment options to slow the progression of AGA or aid hair restoration, selecting the appropriate efficacious treatment with minimal adverse events has always been challenging in both genders. Most of the available treatment options provide only temporary hair growth [7]. While the exact etiopathogenesis of AGA remains unclear, the development of AGA is typically driven by the elevated production of dihydrotestosterone (DHT) hormone [8]. DHT, a potent form of testosterone produced via the action of the 5‐alpha reductase enzyme, binds to the intracellular androgen receptors in the hair follicle dermal papilla cells (DPCs) [9].

Dermal papilla cells (DPCs) are key regulators of the anagen phase of the hair cycle, supplying nutrition and oxygen to hair follicles [10]. When dihydrotestosterone (DHT) binds to androgen receptors in these cells, it triggers follicular miniaturization, ultimately leading to the cessation of hair production [11]. Under low oxygen and limited blood supply, Hypoxia‐Inducible Factor 1α (HIF‐1α) promotes angiogenesis and neovascularization, with its signaling pathway influencing DPC spheroid morphology and regulating hair development and regeneration [12]. However, HIF‐1α is rapidly degraded through hydroxylation mediated by the prolyl‐4‐hydroxylase (P4H) enzyme.

Stemoxydine (L'Oréal, Paris, France) is a competitive P4H inhibitor that may activate HIF‐1α signaling and increase hair density by shortening the kenogen phase, offering a potential treatment for androgenetic alopecia (AGA); however, its therapeutic role remains unconfirmed as no studies have yet evaluated its efficacy in AGA [13, 14]. This study aimed to evaluate the effectiveness of Stemoxydine (Dercos Densi Solution) in adult patients with AGA of varying severity.

## 2 Patients and Methods

### 2.1 Study Design and Setting

This is a prospective pre–post single‐arm exploratory study that was carried out on 35 patients with AGA for 6 months at Outpatient Clinic of Dermatology & Venereology Department of the University Hospital starting from the 1st of July 2023 to the 31st of January 2024 to test the effectiveness of Stemoxydine in managing AGA. The study was conducted to provide preliminary evidence regarding the effects of topical Stemoxydine in AGA.

### 2.2 Participants

Eligible participants were adult patients (≥ 18 years) diagnosed with androgenetic alopecia (AGA). Male patients were graded according to the Norwood–Hamilton scale [15], which ranges from grade 1 to 7 with a type A variant describing anterior involvement, while female patients were graded according to the Ludwig scale, ranging from grade 1 to 3 [16].

Exclusion criteria included those who had received minoxidil therapy within the preceding 3 months, pregnant or breastfeeding women, and women of childbearing potential who were not using adequate contraception. Patients with irregular menstrual cycles, local scalp or hair diseases, systemic diseases known to cause or be associated with AGA, or a history of allergy, anaphylaxis, or hypersensitivity to Stemoxydine were also excluded.

### 2.3 Study Outcomes

The primary outcome of this study was to assess the therapeutic effect of Stemoxydine in treating AGA in both males and females at 6 and 12 weeks using clinical assessment and trichoscopy. The secondary outcomes included adverse events and patient satisfaction.

### 2.4 Evaluation of the Treatment Outcomes

Hair density was measured by using the Dino‐lite Premier AM4113T microscope before the beginning of the treatment, after 6 weeks, and after 12 weeks of final evaluation. Trichoscan analysis software automatically calculated the hair thickness (mm) and hair density (cm^2^). A dermoscopic examination was also performed on the remaining scalp to detect any inflammation, redness, and peripilar signs. These tools enabled objective assessment at fixed scalp points, reducing measurement variability from normal hair‐growth cycle fluctuations.

The evaluation of treatment was made by assessing standardized global photographs taken with a Sony Cybershot camera. The hairs of patients were centered‐parted to take each photographic view. All the photos were registered before entering the study, after 6 and 12 weeks. To limit subjective bias and potential placebo‐related effects, the degree of improvement was graded by two independent and blinded dermatologists who did not participate in the medical care of the patients, both before and after the study period. Clinical improvement was recorded as a subjective score ranging from 0% to 100%, based on a blinded evaluation of the standardized before‐and‐after treatment period. Additionally, they asked each patient about their satisfaction level, categorizing it using a 3‐point Likert scale as unsatisfied, slightly satisfied, or very satisfied.

### 2.5 Study Process and Treatment Regimen

All patients underwent three clinical evaluations: an initial visit, a follow‐up at 6 weeks, and a concluding visit at 12 weeks. Digital photographs, dermoscopic (dermalite 4‐USA) and digital dermoscopic (CompareView Hair version 1.5.06, written by Prof Steven Abbott 2011–13, USA) pictures were captured for every patient before the study and every follow‐up visit under adequate illumination, identical settings, lighting, and position. A measuring tape was used to determine three fixed points on the scalp for dermoscopic evaluation. Dermoscopic readings in the vertex region of the same patients were considered as controls. Following the study's conclusion, two separate dermatologists read each photograph blindly. A third dermatologist concurred with the two primary doctors' contradictory findings. Prescription of Stemoxydine (Dercos Densi Solution) with optimum frequency and dosage for 3 months (application of 2–3 mL of topical Dercos Densi Solution twice daily with gentle massage to the scalp to ensure proper spreading of the solution across the treated scalp area, not as a therapeutic scalp massage intended to increase blood flow or stimulate hair growth). This gentle application technique was not standardized in pressure, duration, or method and therefore does not constitute therapeutic scalp massage.

### 2.6 Sample Size Calculation and Statistical Analysis

The required sample size was calculated using Stata based on the secondary outcome, which was the mean change in hair density (hairs/cm^2^) from baseline to follow‐up. We defined a clinically meaningful improvement as a 4% increase in hair density from baseline, consistent with previously published phototrichogram variability estimates [17]. For an assumed baseline hair density of 120 hairs/cm^2^, a 4% increase corresponds to an absolute mean difference of 4.8 hairs/cm^2^. We assumed a within‐subject standard deviation (SD) of 8 hairs/cm^2^ and a within‐subject correlation (ρ) of 0.30, based on validated phototrichogram reproducibility data [17]. Using a two‐sided significance level of 0.05% and 80% power, the required number of participants was 33. To compensate for an expected attrition rate of approximately 15%, a total of 39 participants was determined to be needed.

### 2.7 Statistical Analysis

The collected data were organized and entered on an Excel sheet and statistically analyzed using SPSS software statistical computer package for Windows, version 25 (IBM Corp., Armonk, NY, USA). The distributional normality of numerical data was assessed using the Shapiro–Wilk test. Accompanying the statistical test, a visualization was created to provide a graphical representation of the data distribution. We performed descriptive statistics to compare the participants' baseline demographics and clinical characteristics. We reported categorical variables as frequency (percent) and compared them using Pearson's chi‐squared (X^2^) test. Continuous variables were presented as mean (standard deviation; SD) or median (interquartile range; IQR) depending on data normality. We compared the before and after values of each endpoint by the end of 3 months after the initial visit to assess the effectiveness of Stemoxydine in treating AGA, utilizing appropriate statistical tests. Mean differences were assessed using the student‐paired *t*‐test or the Wilcoxon signed‐rank test as appropriate. We then performed a sub‐group analysis for the clinical improvement and dermoscopic results to evaluate the differences between males and females. A two‐sided *p* value less than 0.05 was considered statistically significant.

## 3 Results

### 3.1 Demographic and Clinical Data

We enrolled 39 participants. Four patients were lost to follow‐up after the initial visit. The final study cohort included 35 adult patients, 12 males (34.3%) and 23 females (65.7%) (Table 1). The age of patients ranged from 19 to 45 years, with a mean of 29.2 ± 6.98 years. Of the included patients, only 22 (62.9%) had a positive family history of AGA. The severity of alopecia was assessed at the initial visit in both male and female patients, with grades ranging from 2 to 4 in both groups. Among males, most were graded as 3 (50%, *n* = 6), followed by 2 (33.3%, *n* = 4) and 4 (16.6%, *n* = 2). On the other hand, among females, most were graded as 3 (43.47%, *n* = 10), followed by 4 (34.78%, *n* = 8) and 2 (21.7%, *n* = 5) (Table 1).

**TABLE 1:** Baseline demographic and clinical characteristics of study participants.

Parameter	Overall (*n* = 35)	Females (*n* = 23)	Males (*n* = 12)
Age in years, mean (SD)	29.2 ± 6.98	31.1 ± 7.28	25.7 ± 4.87
Positive family history	22 (62.9%)	14 (60.9%)	8 (66.7%)
Alopecia Severity Grade—Grade 2	9 (25.7%)	5 (21.7%)	4 (33.3%)
Alopecia Severity Grade—Grade 3	16 (45.7%)	10 (43.5%)	6 (50.0%)
Alopecia Severity Grade – Grade 4	10 (28.6%)	8 (34.8%)	2 (16.7%)
Hair density (hair/cm^2^)	121.43 ± 14.21	125.30 ± 15.46	114.00 ± 7.41
Hair thickness (mm)	0.01 ± 0.00	0.02 ± 0.00	0.01 ± 0.00

Abbreviations: cm^2^, square centimeter; mm, millimeter; SD, standard deviation.

After 3 months of topical application of Stemoxydine, clinical improvement ranged from 35% to 100%, with a median of 80% (IQR 30%). According to the Quartile Grading Scale, most patients (*n* = 20, 57.1%) showed marked improvement, and 34.3% (*n* = 12) showed moderate improvement (Table 2).

**TABLE 2:** Clinical improvement after 3 months of Stemoxydine treatment.

Characteristics	After 3 months of Stemoxydine application (*N* = 35)
Clinical improvement (%)	
Median (IQR)	80 (30)
Range	35–100
Quartile grading scale, *n* (%)	
Mild	3 (8.6%)
Moderate	12 (34.3%)
Great	20 (57.1)

Abbreviations: IQR, Interquartile range; *N*, total number of included patients; *n* (%), data presented as frequency and percentage.

**FIGURE 1:**
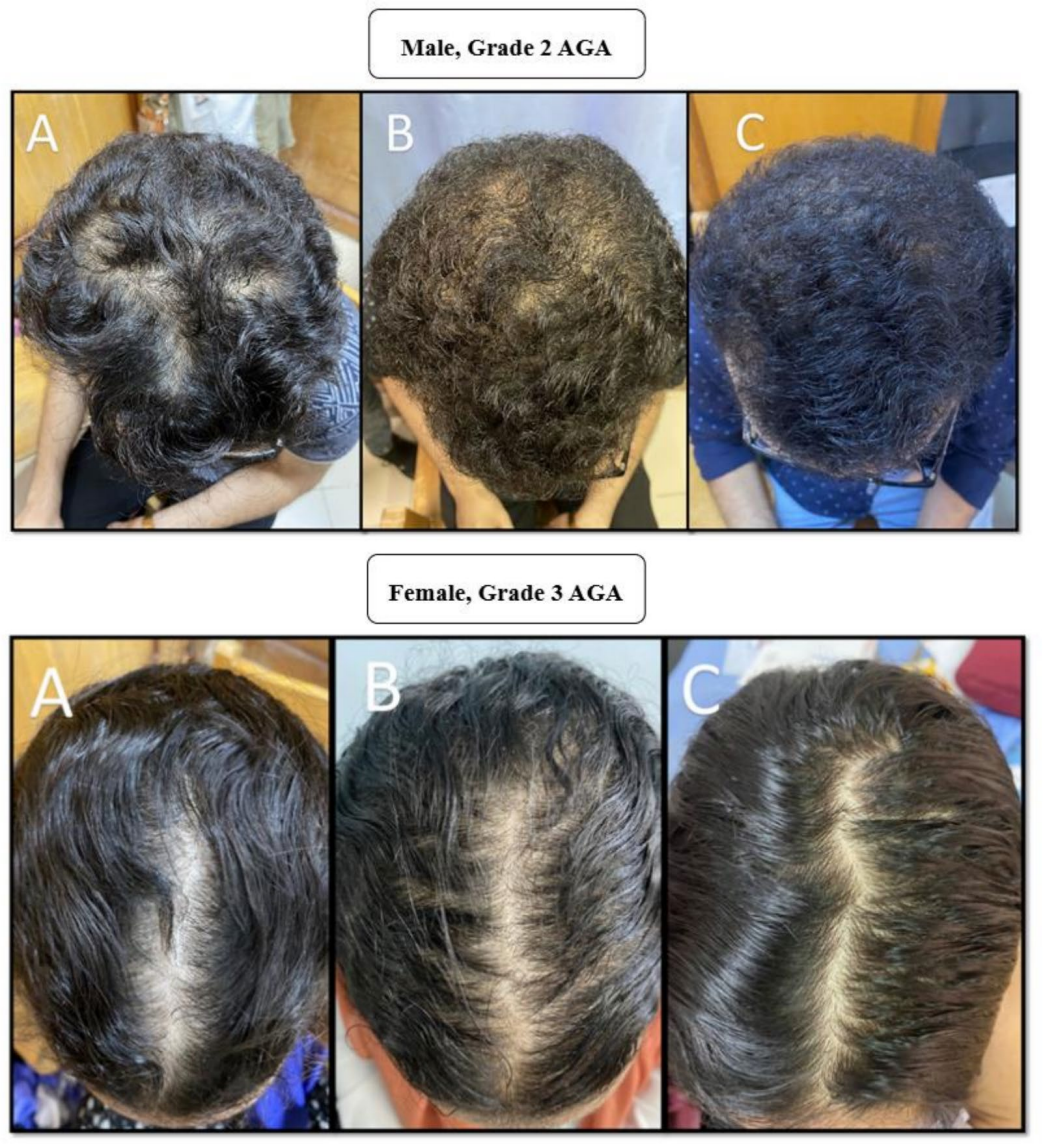
Clinical improvement of vertex region in patients with androgenetic alopecia during Stemoxydine treatment. This figure demonstrates the improvement in hair density in two patients with different grades of androgenetic Alopecia (AGA) over a 3‐month treatment period with Stemoxydine. The top panel showcases a male patient with grade 2 AGA, and the bottom panel depicts a female patient with grade 3 AGA. Each panel contains three images: (A) before the commencement of treatment, (B) 1.5 months following the treatment, and (C) after completing 3 months of treatment.

**FIGURE 2:**
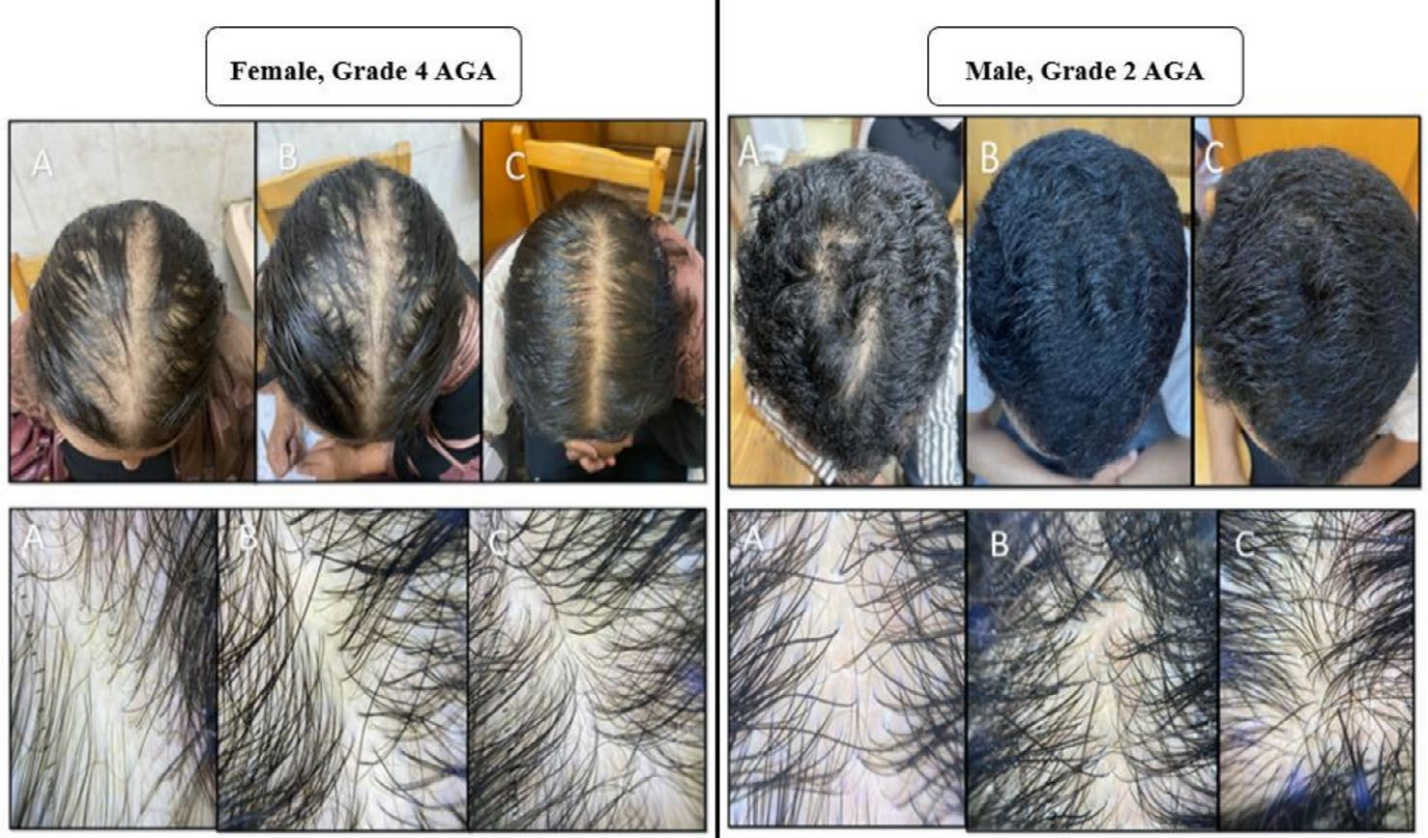
Clinical improvement and dermoscopic results of vertex region in patients with androgenetic alopecia of different grades during Stemoxydine treatment. This figure illustrates the treatment progress in two patients with androgenetic alopecia over 3 months using Stemoxydine. The left panel shows a female patient with grade 4 AGA, and the right panel is a male patient with grade 2 AGA. Each panel contains three images: (A) before the commencement of treatment, (B) 1.5 months following the treatment, and (C) after completing 3 months of treatment. The top rows show the macroscopic changes, while the bottom rows provide a detailed dermoscopic view, demonstrating significant hair regrowth and scalp coverage.

Regarding patient satisfaction, 57.1% (*n* = 20) were satisfied, 28.6% (*n* = 10) were slightly satisfied, and 14.3% (*n* = 5) were not satisfied. The clinical, dermoscopic, and digital dermoscopic assessments revealed varying grades in male and female patients. In males, grades ranged from 0 to 2, with 8.3% graded as 0 (*n* = 1), 66.6% as 1 (*n* = 8), and 25% as 2 (*n* = 3). In females, grades ranged from 0 to 3, with 4.3% graded as 0 (*n* = 1), 13.04% as 1 (*n* = 3), 52.17% as 2 (*n* = 12), and 17.39% as 3 (*n* = 4). A statistically significant difference was observed in alopecia grades before and after 3 months of treatment (*p* < 0.001) (Figures 1 and 2).

### 3.2 Dermoscopic and Digital Dermoscopic Findings

According to dermoscopic examination, new hair growth was observed in 97.1% of patients (*n* = 34) after 6 weeks, with only one patient (2.9%) showing no new hair growth. Regarding digital dermoscopic parameters, hair density at the initial visit before treatment ranged from 90 to 160/cm^2^, with a mean of 121.4 ± 14.21/cm^2^. After 3 months of treatment, hair density ranged from 125 to 252/cm^2^, with a mean of 180.6 ± 46.46/cm^2^. This difference was statistically significant (*p* < 0.001) (Table 2, Figure 1). Hair diameter at the initial visit before treatment ranged from 0.01 to 0.019 mm, with a mean of 0.01 ± 0.002 mm. After 3 months of treatment, hair diameter ranged from 0.016 to 0.029 mm, with a mean of 0.02 ± 0.004 mm. The difference was also statistically significant (*p* < 0.001) (Table 3). Sub‐group analysis comparing clinical improvement and trichoscopy results of males versus females before and after a 3‐month treatment period with Stemoxydine showed significantly higher clinical improvement in males (*p* < 0.001), higher density (*p* < 0.001), and larger diameter (*p* = 0.02) (Table 4, Figures 1 and 2).

**TABLE 3:** Trichoscopy results before and after 3 months of Stemoxydine treatment.

Trichoscopy results	Before treatment	After 3 months	Change from baseline	*p* value
Density (/cm^2^)				**< 0.001** *
Median (IQR)	120 (14)	160 (99)	39 (107)	
Range	90–160	125–252	4–136	
Diameter (mm)				**< 0.001** **
Mean ± SD	0.01 ± 0.002	0.02 ± 0.004	0.006 ± 0.004	
Range	0.01–0.019	0.016–0.029	0.001–0.017	

*Note:* Before means at the initial visit before starting Stemoxydine treatment, while after means at the final visit after 3 months of treatment (applying 2–3 mL of topical Dercos Densi Solution twice daily and gently massaging it into the scalp).

Abbreviations: cm^2^, square centimeter; IQR, Interquartile range; SD, standard deviation.

* Statistical differences between before and after treatment were tested using the Wilcoxon signed rank test. Bold denotes two‐sided statistical significance.

** Statistical differences between before and after treatment were tested using the student‐paired *t*‐test. Bold denotes two‐sided statistical significance.

**TABLE 4:** Subgroup analysis of clinical and trichoscopy results in male vs. female patients before and after 3 months of Stemoxydine treatment.

Characters	Males	Females	*p* value
	(*N* = 12)	(*N* = 23)	
Clinical improvement (%)			< 0.001*
Median (IQR)	96 (19)	70 (20)	
Range	80–100	35–98	
Density (/cm^2^)			
Density after			< 0.001*
Median (IQR)	244 (55)	147 (19)	
Range	140–252	125–250	
Density change			< 0.001*
Median (IQR)	126 (56)	18 (37)	
Range	39–136	4–132	
Diameter (mm)			
Diameter after (mm)			0.14
Mean ± SD	0.02 ± 0.004	0.02 ± 0.004	
Range	0.018–0.028	0.016–0.029	
Diameter change (mm)			0.02**
Mean ± SD	0.008 ± 0.005	0.005 ± 0.004	
Range	0.0–0.02	0.0–0.01	

*Note:* After means at final visit after 3 months of treatment (applying 2–3 mL of topical Dercos Densi Solution twice daily and gently massaging it into the scalp).

Abbreviations: cm^2^, square centimeter; IQR, Interquartile range; mm, millimeter; SD, standard deviation.

* Statistical differences between male and female treatment were tested using the Mann–Whitney *U* test. Bold denotes two‐sided statistical significance.

** Statistical differences between male and female treatment were tested using the student *t*‐test. Bold denotes two‐sided statistical significance.

### 3.3 Adverse Events

Side effects associated with topical Stemoxydine treatment were minimal, primarily manifesting as mild redness, occasional headache, and slight scaling (Table 5).

**TABLE 5:** Side effects associated with Stemoxydine treatment.

	No of patients	%
Hair loss	5	14.3
Redness	8	22.9
Swelling	0	0
Headache	1	2.9
Scales	3	8.6
Allergy	0	0
Hypertrichosis	0	0

## 4 Discussion

In this pre‐post‐study, we assessed the effects of Stemoxydine (Dercos Densi Solution) on AGA in 35 adult patients with varying alopecia grades, applying the treatment twice daily along with scalp massage over 3 months. In our study, participants using Stemoxydine exhibited a median of 80% clinical improvement and more significant increases in hair density compared to baseline with minimal and well‐tolerated adverse events. After 3 months of treatment, clinical improvement assessed by the Quartile Grading Scale showed marked improvement in 57% of patients, moderate improvement in 34.3% of patients, and mild improvement in 8.6% of patients.

Dermoscopic examination revealed new hair growth in 97.1% of patients after 1.5 months of treatment. Digital dermoscopy further demonstrated a significant increase in hair density and diameter after 3 months of topical Stemoxydine treatment compared to baseline. There were statistically significant improvements in AGA grade and increased hair density and diameter, particularly among male participants. Patient satisfaction was high, with 57% expressing satisfaction, 28.6% slight satisfaction, and 14.3% dissatisfaction.

A patented small molecule designed to modulate the hair follicle microenvironment and potentially shorten the kenogen (resting) phase of the hair cycle, has demonstrated some increase in hair density in short‐term clinical studies. Specifically, vehicle‐controlled trials of topical Stemoxydine 5% over 3 months reported a statistically significant increase in visible hair density compared with vehicle control, along with no recorded skin intolerance or significant adverse effects in those studies, suggesting a favorable short‐term safety profile [17]. However, the body of evidence remains limited in scale and scope, with most available clinical data coming from small sample sizes and mostly short durations, and there are no large randomized controlled trials directly comparing Stemoxydine with established FDA‐approved treatments such as minoxidil. Consequently, while early results are promising for modest improvements in hair density and general tolerability, definitive conclusions about the relative efficacy and long‐term safety of Stemoxydine compared with standard AGA therapies cannot yet be drawn. In addition, recent preclinical and early‐clinical studies support the concept of HIF‐1α–mediated follicular reactivation. A recent blinded clinical trial found that a hypoxia‐mimetic topical formulation significantly reduced hair loss and increased hair growth over 6–9 months: responsive subjects showed an average 66.8% reduction in hair loss at 6 months, overall participants had up to 32.5% hair growth by 9 months, with +8.0% increase in anagen hair and −14.0% reduction in telogen hair cycle proportions, along with measurable increases in hair thickness and density (e.g., +7.2% and +14.3%, respectively) [13]. In contrast, a 2023 systematic review of stem cell–based interventions in androgenetic alopecia identified results across 14 studies reporting improvements in hair density with stem cell treatments, but it highlighted substantial heterogeneity in quantitative outcomes and limited sample sizes, calling for larger standardized trials to validate efficacy [18]. Furthermore, a recent comprehensive review of regenerative and stem‐cell–based approaches for AGA identified HIF‐1α modulators and related acellular therapies as promising future directions [18, 19].

However, our results cannot be interpreted as demonstrating equivalence or superiority to minoxidil, as this study lacked a comparator arm. Given the uncontrolled design, the observed improvements should be viewed as preliminary and hypothesis‐generating rather than confirmatory.

These findings are consistent with previous studies comparing a 5% hydro‐alcoholic lotion of Stemoxydine versus a placebo using the phototrichogram technique. The trials involved healthy males aged 18–55 with AGA grades III to V, comprising two intra‐individual studies with product application 5 days a week involving 16 and 23 men, respectively, and an inter‐individual study with daily application on 100 men. Results from the intra‐individual studies demonstrated a significant increase in hair density with Stemoxydine (+4.5%, +11%) compared to the placebo group (−0.3%, +7%) (*p* = 0.04, 0.029, respectively). The inter‐individual study showed an 8% increase in hair density with Stemoxydine versus a 4% change in the placebo group (*p* = 0.036). Data from these trials suggest that Stemoxydine promotes hair density and may shorten the hair kenogen phase [14]. However, the present study does not include a comparator group, and therefore no conclusions can be drawn about comparative efficacy relative to placebo or minoxidil. Despite the variety of treatment options available for AGA, clinicians should consider several factors when choosing treatment, including effectiveness, tolerability, side effects, patient compliance, and cost. The selection of the optimal treatment remains challenging and should be based on personalized therapy [20].

Topical minoxidil is widely recognized as a first‐line treatment for men with mild‐to‐moderate androgenetic alopecia (AGA). It functions by extending the anagen phase and stimulating HIF‐1α, thereby reducing hair loss and enhancing hair growth through increased hair diameter and density [12]. After 16 and 26 weeks of treatment, both concentrations of minoxidil, 2% and 5%, showed a 70% greater improvement in mean hair density compared to placebo [21, 22]. A meta‐analysis confirmed superior outcomes across all concentrations of minoxidil compared to placebo, demonstrating mean differences of 8.11 hairs/cm^2^ and 14.90 hairs/cm^2^ for the 2% and 5% minoxidil treatments, respectively [23]. However, the 5% concentration is associated with higher rates of adverse reactions such as dermatitis, headaches, and hypertrichosis, which can affect treatment adherence, particularly among women [24]. Minoxidil requires a twice‐daily application to the scalp due to its pharmacokinetic properties to effectively stimulate hair growth.

In our study, the reported adverse events were mild and minimal, with no cases of hypertrichosis observed. These findings underscore the effectiveness of Stemoxydine in managing AGA and highlight the need for randomized controlled trials (RCTs) to further explore its pharmacokinetics and compare its effectiveness with FDA‐approved Minoxidil treatments.

## 5 Strengths and Limitations

Overall, this study provided insight into Stemoxydine's effectiveness and its effect on both hair density and diameter for treating AGA. The study involved different grades of AGA in both genders. However, our study had certain limitations. First, the use of a pre–post single‐arm design inherently precludes establishing causality and does not fully differentiate the effects of Stemoxydine from placebo responses or normal fluctuations in the hair‐growth cycle. Second, the 3‐month duration of treatment in this study is shorter than the 6–12 months typically required to fully evaluate sustained therapeutic response in androgenetic alopecia. Although objective tools (Dino‐Lite digital microscopy, Trichoscan software, and dermoscopy) were used to detect early changes in hair density and diameter, the improvements observed may partly represent early, transient telogen–anagen shifts rather than long‐term follicular remodeling. Third, the absence of demographic and patient characteristics variables limits our ability to control for their effects, and future studies should incorporate comprehensive clinical, biochemical, and cosmetic hair assessments to better isolate the true effect of Stemoxydine. The manifestation of these underlying systemic diseases might affect the treatment of AGA. Third, the study duration was limited to 3 months, precluding the assessment of long‐term adverse effects of the drug. Finally, being a single‐center study with a small sample size, our findings may have limited generalizability.

In conclusion, while topical Stemoxydine was associated with measurable improvements in hair density and diameter based on objective assessment tools, these findings must be interpreted within the context of a pre–post single‐arm design that does not establish causality. Our results should be interpreted with caution, as this study was an uncontrolled, short‐term pilot investigation. A RCTs on a wider population is recommended to confirm and expand upon these preliminary results.

## Author Contributions

Yasmina Ahmed Elattar contributed to the conceptualization, methodology, investigation, data curation, and the original draft writing of the study, as well as contributing to the review and editing of the manuscript. Noha Nabil Doghem contributed to the methodology, validation, formal analysis, and provided significant input in the review and editing process of the manuscript. Mai Tarek Amin was involved in the investigation, resource management, data curation, and the visualization of data, alongside assisting in the review and editing of the manuscript. Soha Abdalla Hawwam managed the project administration, coordinated resources, contributed to the review and editing of the manuscript, and was instrumental in providing supervision for the study.

## Funding

The authors have nothing to report.

## Ethics Statement

The study was approved by the Research Ethics Committee of the Faculty of Medicine, Tanta University (code no: 36264PR247/6/23).

## Consent

Prior to participation, written consent was obtained from all patients after providing detailed information about the study's objectives and procedures. Anonymity and confidentiality were maintained throughout the study, including data collection and analysis. All participants signed informed consent forms before starting the study and also for imaging to be published. They were informed of the nature, benefits, and potential risks of their participation in the study.

## Conflicts of Interest

The authors declare no conflicts of interest.

## Data Availability

The data that support the findings of this study are available from the corresponding author upon reasonable request.
